# A Rapid Flp-In System for Expression of Secreted H5N1 Influenza Hemagglutinin Vaccine Immunogen in Mammalian Cells

**DOI:** 10.1371/journal.pone.0017297

**Published:** 2011-02-28

**Authors:** Hanxin Lu, Surender Khurana, Nitin Verma, Jody Manischewitz, Lisa King, John H. Beigel, Hana Golding

**Affiliations:** 1 Division of Viral Products, Center for Biologics Evaluation and Research (CBER), Food and Drug Administration (FDA), Bethesda, Maryland, United States of America; 2 Laboratory of Immunoregulation, Division of Intramural Research, National Institute of Allergy and Infectious Diseases, SAIC-Frederick, NCI-Frederick, Frederick, Maryland, United States of America; University of Georgia, United States of America

## Abstract

**Background:**

Continuing transmissions of highly pathogenic H5N1 viruses in poultry and humans underscores the need for a rapid response to potential pandemic in the form of vaccine. Recombinant technologies for production of immunogenic hemagglutinin (HA) could provide an advantage over the traditional inactivated vaccine manufacturing process. Generation of stably transfected mammalian cells secreting properly folded HA proteins is important for scalable controlled manufacturing.

**Methodology/Principal Findings:**

We have developed a Flp-In based 293 stable cell lines through targeted site-specific recombination for expression of secreted hemagglutinin (HA) proteins and evaluated their immunogenicity. H5N1 globular domain HA1(1-330) and HA0(1-500) proteins were purified from the supernatants of 293 Flp-In stable cell lines. Both proteins were properly folded as confirmed by binding to H5N1-neutralizing conformation-dependent human monoclonal antibodies. The HA0 (with unmodified cleavage site) was monomeric, while the HA1 contained oligomeric forms. Upon rabbit immunization, both HA proteins elicited neutralizing antibodies against the homologous virus (A/Vietnam/1203/2004, clade 1) as well as cross-neutralizing antibodies against heterologous H5N1 clade 2 strains, including A/Indonesia/5/2005. These results exceeded the human antibody responses against the inactivated sub-virion H5N1 vaccine.

**Conclusions/Significance:**

Our data suggest that the 293 Flp-In system could serve as a platform for rapid expression of HA immunogens in mammalian cells from emerging influenza strains.

## Introduction

The recent global spread of swine-origin H1N1 highlighted the need for rapid development of effective vaccines against pandemic influenza viruses. Much of our recent knowledge was derived from studies with the highly pathogenic (HP) H5N1 avian influenza A viruses (AIV) [1]. The H5N1 viruses still cause severe human disease, and may undergo adaptation for human-to-human transmission. As of October 18, 2010 there have been 507 human cases of H5N1 resulting in 302 deaths (fatality rate = 59%) (http://www.who.int/csr/disease/avian_influenza).

Production of hemagglutinin using recombinant technology could overcome the constraints of traditional inactivated influenza vaccine manufacturing that require several months for generation of vaccine viruses using reassortment/reverse genetics, and adaptation for high growth in eggs. Most of the influenza protective antigenic sites are conformation dependent and map primarily to HA1 globular head [2]. In previous reports, codon-optimized HA ectodomain with mutated cleavage site (to prevent processing of HA1-HA2) and an added exogenous foldon sequence at the C-terminus was expressed transiently in 293 cells in order to produce stable oligomers [3], [4], [5]. Technologies that can be easily translated into well controlled large scale manufacturing process will have a great advantage. Thus far, various influenza vaccine prototypes produced in a baculovirus-insect cell expression system have undergone pre-clinical and clinical development [6], [7], but it is not well understood if the baculovirus produced HA products are identical in terms of antigenicity and immunogenicity to the egg grown or mammalian cells based vaccines.

Recombinant proteins produced from mammalian cells are expected to have the same extent of posttranslational modifications as egg grown influenza viruses. Transient transfection of mammalian cells (often HEK293T cells) followed by selection of stable tranfectants, usually result in random integration, clone-to-clone variability and upredicatable level of expression due to position effects based on the site of integration in the host genome. Therefore, one of the important parameters of recombinant protein production for manufacturing is the ability to derive stable mammalian cell lines with defined integration site(s) and reproducible level of protein expression.

Previously, the Flp-In system has been used to express proteins for basic research purposes and in one instance, to produce monoclonal antibodies in CHO cells [8], [9]. In the current study, we have constructed the first vector system for HA protein expression in mammalian cells using the FRT/FLP strategy to overcome position effects and for rapid derivation of stable cell lines expressing HA. As a proof-of-concept, we have cloned the HA0 and HA1 proteins from the highly pathogenic H5N1, A/Vietnam/1203/2004. No codon optimization or modifications to the polybasic cleavage site were made in order to exactly match the HA sequence of the transmitted avian influenza strain [10]. We provide data demonstrating that both proteins are properly folded and can elicit homologous and heterologous H5N1 neutralizing antibodies in rabbits. Importantly, in the face of impending pandemic, cloning, transfection, and stable cell line generation can be done within 2–3 weeks.

## Results

### Cloning of HA1 and HA0 from H5N1 A/Vietnam/1203/2004 in 293Flp-In system

The Flp-In system, which offers a single targeted integration site was found to be more efficient than the conventional transient transfection/selection protocols. Based on a site-specific recombination strategy, a modified Flp-In system was successfully developed to generate stably expressing cell lines. The recombination occurs at a single site and eliminates clonal variability. Expression from the same integration site resulted in reproducible stable levels of insert expression and protein secretion [10], [11]. Therefore such a system is amenable to controlled manufacturing with built in quality assurance steps.

**Figure 1:**
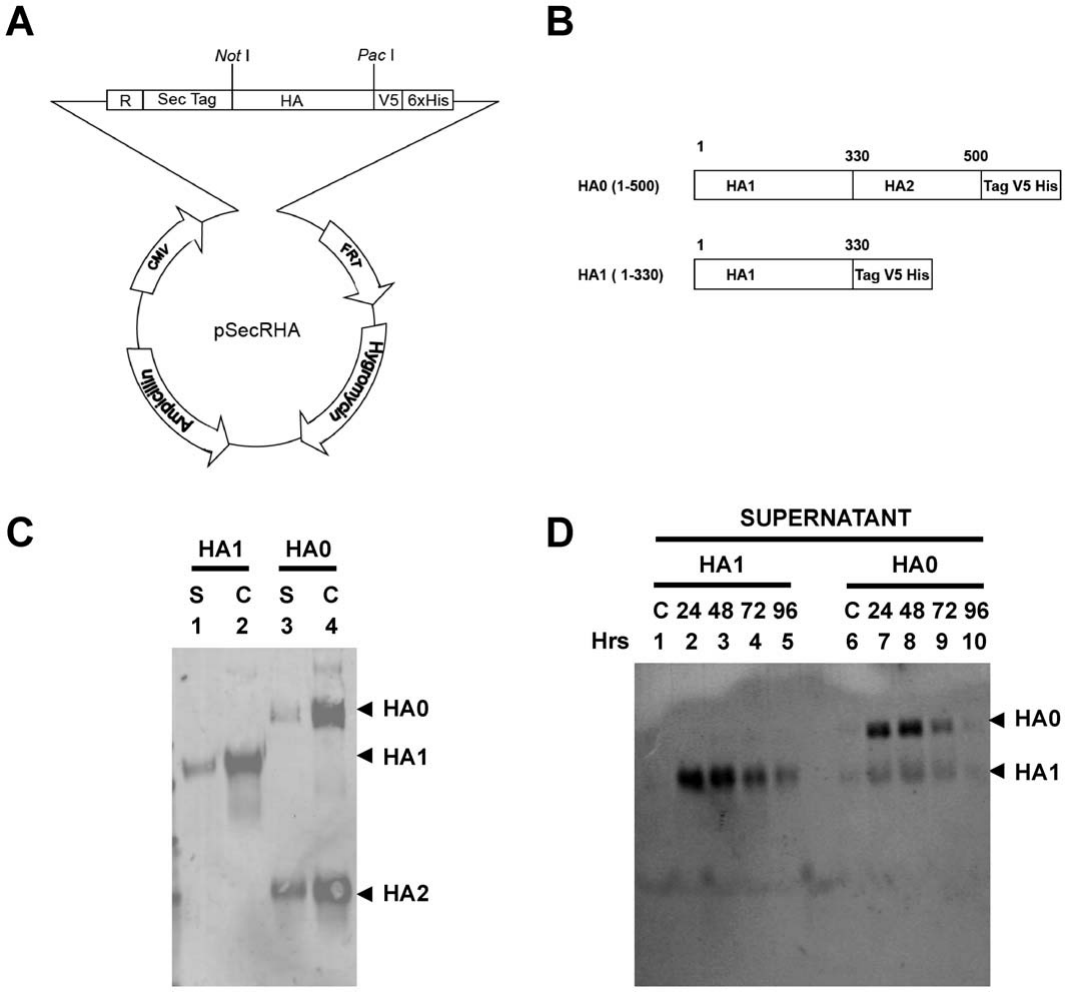
Construction of expression vector for constitutively H5N1 HA secretion in Flp-In based mammalian expression system. (A) To enhance HA protein expression from the FRT-CMV vector (Invitrogen), an RNA splicing sequence from HTLV-I gene and a cassette containing *Not*I and *Pac*I cloning sites was introduced after CMV promoter using Topo cloning system. (B) Schematic of HA1 (1-330) & HA0 (1-500) V5-His_6_ tagged fusion proteins expressed in Flp-In system. The H5N1/Vietnam HA sequence coding for either HA1 (1-330) or HA0 (1-500) was inserted into the vector as a *Not*I*-Pac*I insert. (C–D) Expression and purification of H5N1 A/Vietnam/1203/2004 hemagglutinin proteins from 293 Flp-In stable cell lines. (C) Expression of HA proteins secreted into supernatant from 293 Flp-In cells [S], and in the cell lysates [C] was analyzed by western blot using anti-V5 MAb. (D) HA protein level from supernatant of 293 Flp-In cell culture in serum free medium, collected at different time points: 24, 48, 72 and 96 hours post culture splitting and was analyzed in SDS PAGE followed by western blot with anti-HA1 polyclonal sera.

To enhance HA protein expression and protein secretion from the FRT-CMV vector, a RNA splicing sequence from HTLV-I gene and a cassette containing *Not*I and *Pac*I cloning sites was introduced after the CMV promoter using Topo cloning system, followed by a secretory signal peptide from IgG kappa chain (Fig. 1A). The H5N1 A/Vietnam HA sequences coding for either HA1 (1-330) or HA0 (1-500) were inserted into the vector as *Not*I*-Pac*I inserts. In the case of HA0, the polybasic cleavage site between HA1 and HA2 was not removed or modified, to ensure proper cleavage and folding of the secreted HA0 protein (Fig. 1B). Stable cell clones were selected by growing in DMEM with 150 µg/ml of hygromycin for 10–14 days followed by expansion of individual clones. The selected stable integrants were 100% homogeneous, an important attribute of the Flp-in system. All 40 of selected single–cell clones expressed HA protein at similar levels. For HA protein production, individual cell clones expressing either HA1 globular domain (1-330) or HA0 (1-500) were expanded in T175 flask in serum-free medium for 24 hours. Expression of HA proteins from 293 Flp-In cell lysates (C) and secreted proteins in supernatants (S) were resolved on SDS-PAGE under reducing conditions and detected by Western blot using anti-V5 MAb (Fig. 1C). As expected, under reducing conditions, a significant portion of the cleaved HA0 was separated into HA1 and HA2, only the later reacted with the anti-V5 tag (in the C-terminus of HA2) (Fig. 1C lanes 3, 4). Supernatants were collected at 24, 48, 72 and 96 hours post culture splitting and were analyzed in SDS PAGE followed by western blot with anti-HA1 polyclonal sera. In both HA1 and HA0 expressing cells, the highest level of HA secretion was observed at 24 and 48 hours following cell splitting (Fig. 1D).

In subsequent experiments, H5N1 A/Vietnam/1203/2004 HA1 (1-330) protein and HA0 (1-500) proteins were affinity purified from the 293 Flp-In cell culture supernatant collected 24 hours after culture in serum-free medium using HisTrap column.

### Characterization of purified HA proteins from 293 Flp-In cell

**Figure 2:**
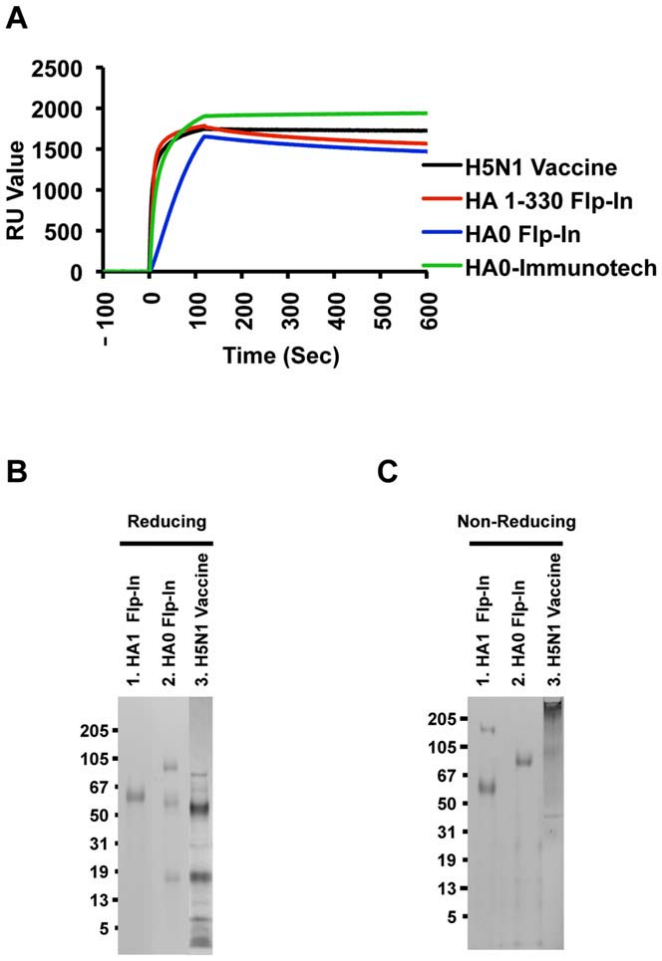
Characterization of purified HA proteins from 293 Flp-In cell. (A) Proper protein folding as demonstrated by steady-state binding equilibrium analysis of conformational dependent human H5N1 neutralizing MAb FLA5.10 (10 µg/ml) to purified Flp-In expressed H5N1 HA1 proteins immobilized on a sensor chip through the free amine group, and onto a blank flow cell, free of peptide. Purified mammalian cell derived H5N1 HA1 or the HA0 proteins obtained from Immune Technology Corp were also analyzed. Binding was recorded using ProteOn system surface plasmon resonance biosensor instrument (BioRad Labs, Hercules, CA). (B–C) Analysis of purified H5N1 protein from Flp-In cells in SDS-PAGE under reducing conditions (B) and non-reducing conditions (C). Purified HA protein from Flp-In cell has higher order protein structure as analyzed by coomassie staining of the reducing SDS PAGE (B) and by coomassie stained non-reducing SDS PAGE (C). Subunit H5N1 vaccine (Sanofi Pasteur) was run as comparator. Western blot analysis of non-reducing SDS PAG using an anti-H5N1 HA1 antibody confirmed the identity of bands observed in coomassie stained gel in Fig. 1C.

Proper protein folding is critical for preservation of HA antigenicity and immunogenicity. Since the majority of influenza neutralizing antibodies recognizes conformational epitopes, we used a panel of human H5N1 neutralizing antibodies generated from B cells of H5N1 A/Vietnam/1203/2004 recovered individuals, that recognized conformational dependent epitopes in HA1 domain of H5N1 A/Vietnam virus [12], [13]. Steady-state binding equilibrium analysis with conformation-dependent human H5N1 neutralizing MAb demonstrated that 293 Flp-In secreted H5N1 HA1 and HA0 proteins were properly folded (Fig. 2A). The licensed inactivated subunit H5N1 vaccine (Sanofi Pasteur) was used as positive controls (Fig. 2A). Similar binding was measured with additional two human MAbs (data not shown).

To determine if the secreted HA1 and HA0 proteins purified from Flp-In cells form higher order quaternary forms, they were analyzed in SDS-PAGE under reducing vs. non-reducing conditions. For comparison, subunit H5N1 vaccine (Sanofi Pasteur) was also subjected to analysis. The Flp-In derived HA0 ran as a single band under non-reducing conditions, but was also present in cleaved form that separated into HA1 and HA2 under reducing conditions (Fig. 2B and 2C, lane 2). Unexpectedly, the Flp-In derived HA1 (1-330) contained both a monomer band and higher MW species under non-reducing conditions, (Fig. 2C, lane 1). Western blot confirmed that both bands are reactive with rabbit anti HA sera. Subunit H5N1 HA vaccine ran primarily as oligomers in non-reducing gel, which dissociated into HA1 and HA2 forms under reducing conditions (Fig. 2B and 2C, lane 3).

**Figure 3:**
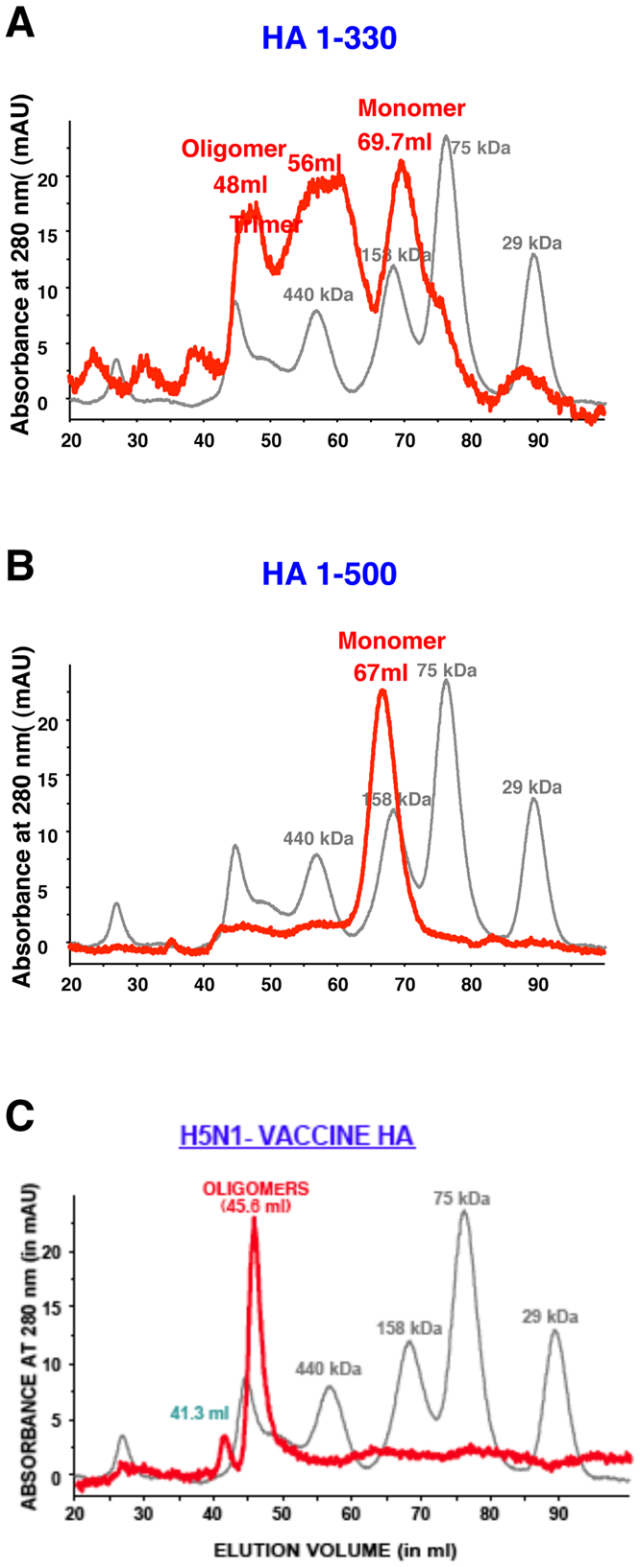
Analysis of HA protein by gel filtration chromatography. Superdex S-200 gel filtration chromatography of Flp-In derived H5N1 HA proteins. Purified H5N1 HA1 (1-330) protein (A) or the HA0 (1-500) protein (B) were subjected to gel filtration. The panels present superimposed elution profiles of purified HA proteins (blue line) overlaid with calibration standards (grey line). The elution volumes of protein species are shown in parenthesis. While purified HA 1-330 protein was presented in monomer, trimer and oligomer form (A), the HA1-500 protein was observed primarily in a monomeric form (B). Gel filtration profile of Subunit H5N1 vaccine (Sanofi Pasteur) (C) show predominance of oligomers.

To better decipher the quaternary forms in the Flp-In derived HA1 and HA0, we subjected the purified HA proteins to Superdex S-200 gel filtration chromatography. While the HA0 (1-500) protein was predominantly monomeric (Fig. 3B), the HA1 (1-330) protein contained monomers and higher MW oligomers (Fig. 3A). In comparison, the subunit H5N1 HA vaccine contained predominantly oligomers (Fig. 3C).

### Flp-In derived proteins elicit H5N1-specific broadly neutralizing antibodies in Rabbits

**Figure 4:**
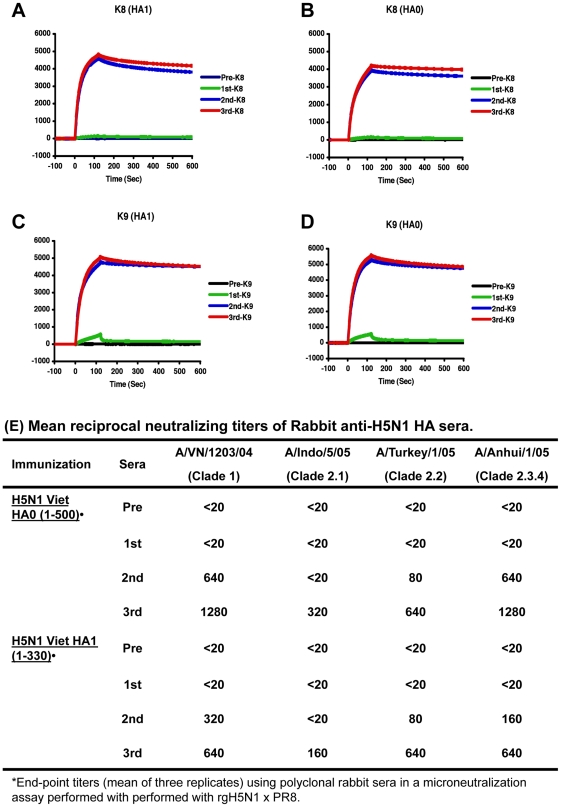
Development of neutralizing and anti-HA binding antibodies in rabbits following vaccination with H5N1 HA1 and HA0 proteins produced using Flp-In system. (A–D) Antibody kinetics following H5N1 HA1 & HA0 vaccination in rabbits. Steady-state binding equilibrium analysis of pre & post-H5N1 HA1 immune sera (rabbit K8) in (A–B) or pre- & post-H5N1 HA0 immune sera (rabbit K9) in (C–D) to Flp-In derived H5N1 HA1 and HA0 proteins were measured using SPR. Ten-fold diluted individual post-vaccinated sera from each time point, were injected simultaneously onto recombinant Flp-In H5N1 HA1 in (A and C) and H5N1 HA0 in (B and D), immobilized on a sensor chip through the free amine group, and onto a blank flow cell, free of peptide. Binding was recorded using ProteOn system surface plasmon resonance biosensor instrument (BioRad Labs, Hercules, CA). (E) Flp-In HA1 and HA0 elicit high cross-neutralizing antibody titers against homologous and heterologous H5N1 viruses. Animals were immunized with 100 mg proteins mixed every three weeks. Sera was collected at 8^th^ day after each vaccination and analyzed in a microneutralization assay against various H5N1 virus strains. Data is representative of three experiments.

New Zealand rabbits were immunized thrice intra-muscularly at 21-days interval with 100 µg of purified HA1 and HA0 proteins based on the currently recommended human dose of 90 µg HA for the licensed unadjuvanted subunit H5N1 vaccine for pre-pandemic use in US [1]. Total binding to either HA1 or HA0 was measured after each vaccination using SPR. At least two doses of the recombinant HA proteins were required to elicit antibody response, as previously described for the inactivated H5N1 vaccine [1]. Post-second and third vaccination sera from rabbits immunized with either HA1 or HA0 derived from the 293 Flp-In system bound with high avidity to both HA1 and HA0 proteins (Fig. 4 A–D). In the microneutralization assays, the rabbit sera after two immunizations with the H5N1 HA0 protein elicited very significant neutralizing antibody titers against both homologous (A/Vietnam/1203/2004, clade 1) and heterologous strains (A/Turkey/1/05, clade 2.2 and A/Anhui/1/05, clade 2.3.4). After the third vaccination these titers further increased and cross neutralization of H5N1 A/Indonesia/5/05 (clade 2.1) was also observed (Fig. 4E). Interestingly, the HA1 globular domain protein generated similar titers of neutralizing antibodies with the same profile of cross-clade neutralizing activity as was observed for the intact HA0 protein (two-fold difference in titers falls within assay variability). Similar results were obtained with the licensed inactivated H5N1 subunit vaccine administered to rabbits at 100 mg dose mixed with the TiterMax adjuvant. After the second and third vaccination a titer of 2560 against A/Vitenam/1203/04 was observed with modest cross reactivity against A/Indonesia/05/2005 (clade 2.1), A/Turkey/1/05 (clade 2.2), and A/Anhui/1/05 (clade 2.3.4).

For comparison we measured neutralizing titers in the plasma of humans enrolled in a clinical trial, using the monovalent inactivated subunit A/Vietnam/1203/04 H5N1 vaccine (Sanofi Pasteur) that has subsequently been licensed. As previously described, increasing doses of vaccine (90, 120, 180 µg of HA), were administered to study participants on days 0, 28, 56, and 84, for a total of four vaccinations [14]. We have expanded the previously published microneutralization assays to include several heterologous H5N1 strains from clade 2 sub lineages (Table 1). Even after three immunizations the neutralization titers in humans against the homologous strain were lower than what was observed with the rabbit sera. In addition, minimal if any cross neutralization of heterologous strains was measured irrespective of the vaccine dose even after three immunizations (Table 1). The differences in the vaccination outcome between rabbits and humans with the inactivated H5N1 subunit vaccine may reflect the lower body mass and/or the addition of the TiterMax (water in oil) adjuvant. Several clinical trials demonstrated that oil-in-water adjuvants such as MF59 and AS03 greatly enhanced the immunogenicity of inactivated H5N1 vaccines in human populations [13], [15], [16], [17].

**Table 1:** Mean Reciprocal Neutralizing Titers of Post-H5n1 Human Sera.

SAMPLE	VIETNAM	TURKEY	ANHUI	INDONESIA
	(CLADE 1)	(CLADE 2.2)	(CLADE 2.3.4)	(CLADE 2.1)
**90 µg H5N1 Vaccine**				
**1**	40	<20	20	<20
**2**	40	20	40	<20
**3**	**<20**	<20	<20	<20
**4**	**20**	<20	<20	<20
**120 µg H5N1 Vaccine**				
**1**	40	20	40	<20
**2**	40	<20	20	<20
**3**	**80**	**20**	40	<20
**4**	**20**	<20	20	<20
**180 µg H5N1 Vaccine**				
**1**	**40**	<20	20	<20
**2**	80	20	40	<20
**3**	**80**	**40**	40	20
**4**	**40**	**20**	**20**	<20

*End-point titers (mean of three replicates) using human sera after two vaccinations with inactivated H5N1 vaccine in a microneutralization assay performed with performed with rgH5N1 x PR8.

These data confirmed that the 293 Flp-In derived proteins were properly folded, presented relevant conformational epitopes, and were immunogenic in rabbits. The ability to elicit cross neutralizing antibodies against clade 2 viruses, including A/Indonesia/5/2005 (clade 2.1) is very important, since it is unknown which H5N1 strain will adapt to bind better to the human α2-6 sialic acid receptor, resulting in human to human transmission.

## Discussion

Recombinant protein technology may provide a rapid response to emerging influenza infections with pandemic potential. The current vaccine campaign is based on a traditional process that depends on the derivation of reassortant viruses in mammalian cells followed by adaptation to high growth in eggs, which may result in mutations in the key antigenic sites. In addition, vaccine potency reagents require repeat vaccinations of sheep with bromelain cleaved HA from egg-grown vaccine strains. Therefore, recombinant technology can alleviate some of the bottlenecks and assist in production of early vaccines or potency reagents for vaccine lot release. Transient expression or random integration based cell lines are widely used for protein expression, but these technologies have several significant shortcomings including time-consuming, unpredictable and unreproducible levels of gene expression primarily due to lack of control over the position of integration. To that end, it will be important to use a certified mammalian cell line with a built in genomic site specific recombination cloning site and an optimized expression vector that can be used within short time frame for cloning and selection of stable integrants secreting properly folded HA proteins [18].

In the current study we provide the first data supportive of the use of 293 Flp-In based stable mammalian system for influenza hemagglutinin expression. The main findings were (a) Both HA1 and HA0 proteins from H5N1 HP A/Vietnam/1203/2004 were successfully expressed in 293 Flp-in cells and stable integrants were selected within 10-14 days; (b) the secreted HA1 and HA0 were purified from the supernatants of cells growing in serum free conditions and were shown to be properly folded as determined by binding to conformation dependent human MAbs; (c) the HA0 with intact polybasic cleavage site contained cleaved (HA1 and HA2) as well as uncleaved molecules (similar to HA on virions); (d) rabbit vaccination with the HA1 and HA0 proteins resulted in homologous virus-neutralizing antibodies with a significant cross neutralization of heterologous strains representing recently transmitted H5N1 clades; and (e) sera from humans vaccinated three times with the licensed H5N1 A/Vietnam/1203/2004 inactivated vaccine had lower titers against the homologous virus and negligible neutralizing antibody titers against heterologous H5N1 strains from multiple clades.

The use of site-directed recombinant technology for HA production in stable mammalian expression cell lines eliminates the handling of live highly pathogenic H5N1 viruses. It also circumvents the need for generation of egg-adapted high growth virus in the face of impending pandemic. High-level protein expressing mammalian cell lines could be reproducibly constructed based on the exchange of HA expression cassettes in a pre-tagged desired locus of the engineered cell line by site-specific recombination.

Our data supports and extend the findings of Wei et al. [3] and Bosch et al. [5] who used transient expression systems for expression of HA proteins. In this study, both secreted HA1 and HA0 expressed in the Flp-In system in the same integration site and were purified from the culture supernatants. The proteins were properly folded and were recognized by conformation dependent H5N1 neutralizing human MAbs.

Following vaccination, the kinetics of HA-binding antibody development (measured by SPR) paralleled the appearance of potent broadly neutralizing antibodies confirming the immunogenicity of Flp-in derived secreted HA1 and HA0 proteins. From the rabbit studies, the Flp-In derived HA1 (1-330) protein seems as immunogenic as intact HA0 (1-500) in generation of broadly H5N1 neutralizing antibodies and might be a promising vaccine candidate.

Importantly, the neutralization titers and breadth of cross clade neutralization observed in the current study using recombinant HA proteins, were higher than the neutralization titers measured in the sera of human volunteers vaccinated with escalating doses of the currently licensed H5N1 inactivated vaccine. Suguitan *et al.* previously demonstrated that restoring the polybasic cleavage site in the H5 HA of the vaccine virus improved its immunogenicity and efficacy, suggesting that removal of polybasic cleavage site due to safety concerns, had a deleterious effect on the immunogenicity and efficacy of the H5N1 HA [19]. Therefore, it is plausible that the HA0 with intact polybasic cleavage site expressed in Flp-In system, which was cleaved into HA1 and HA2 forms provided better immunogenicity than what has been earlier reported for HA with mutated cleavage site [3].

In the face of impending pandemic, generation of vaccine in a timely manner is critical. While adjuvants were shown to improve the immunogenicity of inactivated vaccines against H5N1 and SOIV-H1N1 [13], [15], [16], [17], there is still a considerable time gap between virus isolation and generation of reassorted vaccine seed, as was evident during the recent SOIV-H1N1 pandemic. Therefore, recombinant technologies may provide an important early vaccine response alternative. The Flp-In system provides an advantage over the transient transfection approach.

Based on these data it will be prudent to derive a 293 Flp-In system that can be used for production of HA proteins for vaccine generation as well as reagents that can be used for quality assurance and vaccine release purposes. In addition to human vaccines, poultry vaccination may become an important early approach for curtailing the spread of newly emerging HP viruses and their transmission to susceptible human populations [20], [21], [22], [23].

## Materials and Methods

### Cloning and protein expression

A pSecRTag vector was modified to incorporate a HTVL-I splice site (R) using Topo cloning system (Invitrogen) into the pSecTag vector (Invitrogen). This vector further modified that incorporated the *Not*I and *Pac*I cloning site between the splice site and the V5-His_6_ tag for cloning of desired genes (Fig. 1A). A cDNA corresponding to residues HA1-330 or HA1-500 of the hemagglutinin (HA) from A/Vietnam/1203/2004 (GenBank accession no. GI:50296053) was cloned into the pSecRTag vector using *Not*I and *Pac*I cloning site. The expressed fusion protein contains V5-tag and His_6_-tag residues at the C terminus (GKPIPNPLLGLDSTRTGRTGHHHHHH), and IgK-chain secretion signal peptide at the N terminus (Fig. 1B).

### Protein expressing and purification

Plasmids expressing a secretory HA and a Flp recombinase were transfected into the human embryonic kidney cell line 293 Flp-In cell (Invitrogen, Carlsbad, CA) using lipofectamine 2000 reagent (Invitrogen, Carlsbad, CA). At the day of transfection, DMEM medium was replaced with fresh medium. One mg of HA expression plasmid pSecRHA and 9 mg of pOG44 were co-transfected using lipofectamine 2000 reagent. Twenty-four hours after transfection, cells were grown in the fresh DMEM medium containing 150 µg/ml of hygromycin. The medium was replaced every three days. Ten days after selection, about 40 cell clones were selected for protein expression analysis. For control, mock transfected cells were all killed by hygromycin. Individual clones were expanded and maintained in DMEM with hygromycin.

For HA protein production, cells expressing either HA(1-330) or HA(1-500) were grown in T175 flask in serum-free medium and incubated for 24 hours. Then HA was purified from the cell supernatant by metal affinity chromatography using HisTrap columns (GE Healthcare, Piscataway, NJ). Fractions containing HA proteins were combined and desalted using PD10 column (GE Healthcare, Piscataway, NJ). The expression of the HA proteins was confirmed by sodium dodecyl sulfate-polyacrylamide gel electrophoresis (SDS-PAGE) under reducing and non-reducing conditions and western blotting using a mouse monoclonal anti-V5 antibody (Invitrogen), or a rabbit polyclonal anti-H5N1 HA1 specific sera.

### Gel filtration chromatography

Proteins at a concentration of 5 mg/ml were analyzed on Superdex S200 XK 16/60 column (GE Healthcare, Piscataway, NJ) pre-equilibrated with PBS, and the protein elution was monitored at 280 nm. Protein molecular weight marker standards (GE healthcare) were used for column calibration and generation of standard curve to identify the molecular weights of the test protein sample.

### Protein conformation analysis by surface plasmon resonance

Steady-state equilibrium binding of conformation dependent human H5N1 neutralizing MAb FLA5.10 was monitored at 25°C using a ProteOn surface plasmon resonance biosensor (XPR36, BioRad Labs). The HA proteins were coupled to a GLC sensor chip using amine coupling with 100 resonance units (RU) in the test flow cells. Samples of freshly prepared FLA5.10 human monoclonal antibody at various concentrations were injected at a flow rate of 30 µl/min (120-s contact time). Flow was directed over a mock surface to which no protein was bound, followed by the HA proteins coupled surface. Responses from the peptide surface were corrected for the response from the mock surface and for responses from a separate, buffer only, injection. MAb 2D7 (anti-CCR5) was used as a negative control antibody in various binding experiments. Binding Kinetics for the MAbs and the data analysis was performed using BioRad ProteON manager software (version 2.0.1).

### Rabbit immunization

New Zealand rabbits were immunized thrice intra-muscularly at 21-days interval with 100 µg of purified HA proteins mixed with TiterMax adjuvant.

### Microneutralization assay

Viral-neutralizing activity was analyzed in a microneutralization assay based on the methods of the pandemic influenza reference laboratories of the Center for Disease Control and Prevention (CDC). Low pathogenicity H5N1 viruses, generated by reverse genetics, were obtained from CDC: A/Vietnam/1203/2004 (SJCRH, clade 1), A/Indonesia/5/2005 (PR8-IBCDC-RG2; clade 2.1), A/Turkey/1/05 (NIBRG-23; clade 2.2), A/Anhui/1/05 (IBCDC-RG5, clade 2.3.4). The experiments were conducted with three replicates for each serum sample and performed at least twice.

### Microneutralization titers in human sera vaccinated with the licensed inactivated H5N1 vaccine

This study was conducted by the NIAID, NIH. Inactivated subvirion H5N1 vaccine (rgA/Vietnam/1203/04 X A/PR/8/34) that contains 90 µg/ml HA was used as a vaccine (*Sanofi Pasteur*). A single-center dose escalating unblinded clinical trial was conducted to determine if it is possible to increase the HAI and neutralizing antibody titers against avian influenza by additional boosts and/or increasing the vaccine dose beyond 90 µg HA [14]. The study was conducted under NIH IRB approval (Clinical Trials.gov identifier NCT00383071). Post vaccination sera samples were evaluated in the microneutralization assays (under FDA/NIH Research Involving Human Subjects exemption 03-118B), using multiple low pathogenicity H5N1 viruses generated by reverse genetics as previously described [13].

### Ethics Statement

All procedures were in accordance with the National Research Council (NRC) Guidelines for the Care and Use of Laboratory Animals, the Animal Welfare Act, and the Centers for Disease Control (CDC)/National Institutes of Health (NIH) Bio-Safety Guidelines in Microbiological and Biomedical Laboratories and approved by the Institutional Animal Care and Use Committee (IACUC). The study was conducted under National Institutes of Health (NIH) IRB approval (Clinical Trials.gov identifier #NCT00383071).
